# Epilepsy Surgery in a Resource-Limited Latin-American Center: Presurgical Evaluation Findings and Early ILAE Outcomes in a Mixed Adult-Pediatric Cohort

**DOI:** 10.3390/brainsci16070729

**Published:** 2026-07-09

**Authors:** Fabrizio A. Mortola, Ilse M. Mora-Rodríguez, Juan C. Barrera de Leon, Tania P. Sánchez-Murguía, Brenda Vega-Ruiz, Jonathan A. Cisneros-Orozco, Marco A. Román-Delgadillo, Andrea Enríquez-Zaragoza, Karla López-Jiménez, Mario A. Alonso-Vanegas, Fridha V. Villalpando-Vargas, Alioth Guerrero-Aranda

**Affiliations:** 1 Los Valles University Center, University of Guadalajara, Ameca 46600, Mexico; fabrizio.mortola@academicos.udg.mx (F.A.M.); ilse.mora@academicos.udg.mx (I.M.M.-R.); juan.bdeleon@academicos.udg.mx (J.C.B.d.L.); tania.sanchez@academicos.udg.mx (T.P.S.-M.); brenda.vega@academicos.udg.mx (B.V.-R.); karla.lopez@academicos.udg.mx (K.L.-J.); viridiana.villalpando@academicos.udg.mx (F.V.V.-V.); 2 Epilepsy Clinic, Hospital “Country 2000”, Guadalajara 44610, Mexico; jonathan.cisneros4792@alumnos.udg.mx (J.A.C.-O.); marcoroman2899@gmail.com (M.A.R.-D.); andyferzara94@gmail.com (A.E.-Z.); 3 International Center for Epilepsy Surgey, Humanitas Medical Group (HMG), Mexico City 04380, Mexico

**Keywords:** epilepsy surgery, temporal lobectomy, focal cortical dysplasia, hippocampal sclerosis, corpus callosotomy, low- and middle-income countries

## Abstract

**Highlights:**

**What are the main findings?**

- Structured presurgical evaluation enabled safe epilepsy surgery selection in a resource-limited Latin-American center (*n* = 22).
- Curative-intent procedures achieved 60% seizure freedom (ILAE I), with no deaths or permanent neurological deficits.

**What are the implications of the main findings?**

- Effective epilepsy surgery programs are feasible in low-resource settings without advanced infrastructure.
- Outcome differences across procedure groups reflect surgical intent (curative vs. palliative), not procedure effectiveness.

**Abstract:**

Background: Resource-constrained programs perform epilepsy surgery under limited access to advanced imaging and neuromodulation. We describe presurgical evaluation findings, early seizure outcomes, and safety from a mixed adult-pediatric cohort. Methods: Retrospective single-center series of 22 consecutive patients meeting surgical candidacy. We captured demographics, epilepsy classification/etiology, presurgical investigations (long-term Video-EEG, MRI, selective FDG-PET), procedure type, histopathology when available, and postoperative seizure outcome (ILAE). Complications were recorded using the ILAE adverse-event taxonomy. Results: Mean age at surgery was 21.2 ± 11.2 years; 15 (68%) were male. Epilepsy was focal in 15 (68%); structural etiologies predominated in 15 (68%). MRI was concordant with the presumed epileptogenic zone in 13 (59%). FDG-PET was obtained in 10 (45.5%) and was concordant in 7 (70%). Long-term Video-EEG (≥2 habitual seizures) was completed in 21. Mean delay to surgery was 10 years (IQR [8–15]); presurgical work-up averaged 10 months (IQR [6–15]). Procedures were resective 14 (64%), disconnective 6 (27%), and neuromodulatory 2 (9%). Histopathology was available in 16 cases, most commonly showing hippocampal sclerosis (*n* = 5) and focal cortical dysplasia (*n* = 5). At 14 months median follow-up (range 12–34), ILAE outcomes were: I 41% (9), II 14% (3), III 23% (5), IV 23% (5). Outcomes significantly differed by procedure: curative-intent (resection/disconnection) achieved ILAE I 60% (9) versus ILAE III/IV 7 after palliative-intent (corpus callosotomy/neuromodulation). No deaths or permanent deficits occurred; one corpus callosotomy case developed transient aseptic meningitis. Conclusions: In a resource-limited program, structured presurgical evaluation and careful selection yield resection/disconnection outcomes comparable to high-resource benchmarks, while corpus callosotomy/neuromodulation remain largely palliative. Practical, reproducible pathways may help shorten delays and improve access in similar settings.

## 1. Introduction

Epilepsy is one of the most common serious neurologic disorders worldwide, affecting an estimated 45–50 million people [1]. Despite the availability of numerous anti-seizure medications, a substantial subset of patients experience ongoing seizures that are drug-resistant. Approximately one-third of individuals with epilepsy do not attain seizure control with optimal medical therapy [1]. Moreover, in some specific etiologies (e.g., focal cortical dysplasia), up to 75% of patients may develop drug-resistant epilepsy (DRE) [2]. These cases of DRE carry a tremendous burden in terms of disability, injury risk, and mortality [3]. Notably, the risk of premature death is markedly elevated in DRE. One report observed that as many as 12% of patients died within two years of a DRE diagnosis if seizures remained uncontrolled [4]. This underscores the urgent need for more effective interventions for patients whose epilepsy cannot be managed with pharmacotherapy alone.

For selected patients with DRE, surgical intervention offers a proven therapeutic avenue that can significantly improve outcomes. In properly chosen candidates, epilepsy surgery can achieve long-term seizure freedom in a large proportion of cases, which in turn improves quality of life and other functional domains [5]. A randomized trial in patients with temporal lobe epilepsy demonstrated the clear superiority of surgical treatment over continued medical management, with 58% of surgical patients becoming seizure-free at one year compared to only 8% of those on medication [6].

Numerous observational studies and meta-analyses have likewise shown that roughly 60–70% of patients undergoing resective surgery for focal DRE achieve freedom from disabling seizures, whereas persistent seizures are the norm with ongoing medical therapy [5,7,8,9]. Accordingly, international guidelines advocate early evaluation for surgical eligibility in suitable DRE patients, as surgery not only improves seizure control but can also reduce DRE-associated morbidity and mortality [10].

Despite the well-established benefits of epilepsy surgery, there remains a stark global disparity in access to this life-changing treatment. Nearly 80% of people with epilepsy reside in low- and middle-income countries (LMICs), yet the vast majority of specialized epilepsy surgery centers and resources are concentrated in high-income regions [3]. In fact, many LMICs have no active epilepsy surgery program at all, and those that do exist often have limited capacity. This imbalance has created a large “surgery gap” between the number of patients who could potentially benefit from surgery and those who actually receive it [11,12,13].

The consequence is that DRE patients in resource-limited settings often endure prolonged, inadequately controlled epilepsy for years or even decades [14,15]. For example, one multicenter study reported a mean delay of about 20 years from epilepsy onset to surgical intervention among adult patients [16]. Addressing this inequity in care is a critical public health challenge. There is a pressing need to raise awareness about the role of surgery in improving outcomes for DRE and to expand access to surgical options in resource-limited settings.

In this context, we report a case series from a recently launched epilepsy surgery program, aiming to illustrate the impact of surgical treatment in a resource-limited setting. This cross-sectional analysis of patients who underwent epilepsy surgery between 2022 and 2024 (with a minimum one-year follow-up) highlights the outcomes and challenges in our cohort. Our experience adds to the growing evidence that epilepsy surgery is both feasible and beneficial in low-resource environments, and underscores the imperative of expanding access to surgical care for DRE patients in underserved regions.

## 2. Materials and Methods

### 2.1. Study Design and Setting

We conducted a retrospective observational cohort study with longitudinal postoperative follow-up, including all consecutive patients who underwent epilepsy surgery within the Epilepsy Surgery Program at the Epilepsy Clinic, Hospital Country 2000 (Guadalajara, Mexico), between 1 February 2022 and 31 December 2024. The program serves a mixed adult-pediatric population referred from western Mexico and functions in a low- and middle-income environment with limited specialized resources. The study followed the Declaration of Helsinki and was approved by the Los Valles University Center Ethics Committee (reference CEI/56/2024) on 2 April 2025. All patients or guardians gave written informed consent for surgery and secondary use of de-identified data.

### 2.2. Participants

Inclusion criteria included: (1) drug-resistant epilepsy (failure of ≥2 appropriately chosen and tolerated antiseizure medications); (2) completion of the center’s standard presurgical protocol (long-term video-EEG, high-resolution MRI, and, when indicated, FDG-PET, neuropsychology); (3) surgical intervention during the study window; (4) minimum documented post-operative follow-up of ≥12 months. Patients with incomplete data, prior epilepsy surgery elsewhere, or lost to follow-up before one year were excluded.

### 2.3. Variables and Data Analysis

For each patient, we extracted: demographic data, epilepsy etiology, seizure semiology, lateralization/localization, concordance of ancillary studies (defined as agreement between presurgical test findings and the presumed epileptogenic zone as confirmed by postoperative seizure outcome), surgical procedure, histopathology (when requested), and post-operative seizure outcome (ILAE classification) [17]. Complications were recorded according to the ILAE adverse-event taxonomy [18]. Dates of initial diagnosis, first evaluation at our center and definitive surgery were used to calculate the “delay to surgery” and “presurgical work-up time”. See Table 1.

Data were anonymized and entered into an electronic spreadsheet (Microsoft Excel) and analyzed with R v4.3. Descriptive statistics are presented as mean ± standard deviation (SD) or median interquartile range (IQR) for continuous variables and frequency (percentage) for categorical variables. Between-group outcomes comparisons (resective vs. disconnective) employed the Kruskal–Wallis test; *p* < 0.05 was considered significant.

## 3. Results

### 3.1. Study Cohort

Twenty-two patients met the inclusion criteria (Table 1). The mean age at surgery was 21.2 ± 11.2 years and 68% were male. Fifteen cases (68%) had focal epilepsy and seven (32%) had generalized or multifocal epilepsies. Structural etiologies predominated (*n* = 15, 68%), followed by unknown (*n* = 6, 27%) and autoimmune (*n* = 1, 5%).

**Figure 1:**
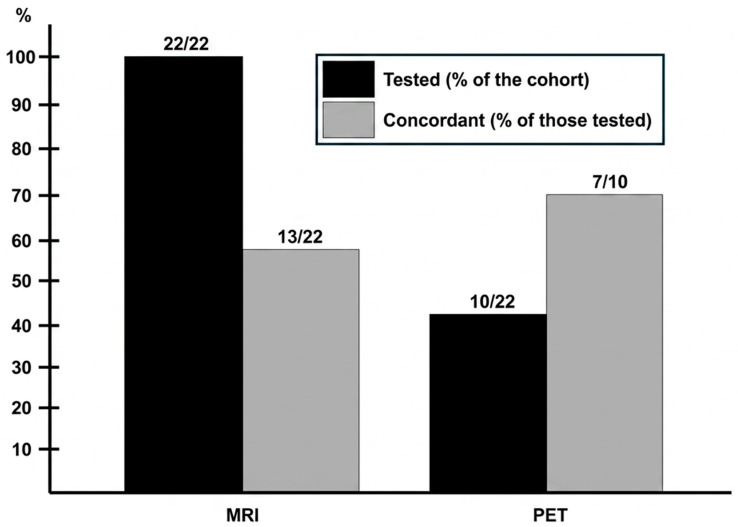
Imaging utilization and concordance. Bars display the proportion of the cohort undergoing each test (MRI 22/22; PET 10/22) and concordance among those tested (MRI 13/22 = 59%; PET 7/10 = 70%). Concordance is calculated using the number tested as the denominator.

MRI was concordant with the presumed epileptogenic zone in 13 patients (59%). FDG-PET was obtained in ten patients (45.5%) belonging to the structural etiology category, and was requested selectively when additional functional data were expected to strengthen presurgical localization. PET was concordant in seven patients (70% of PET studies) (see Figure 1).

All but one patient completed successful (at least two seizures captured) long-term video-EEG monitoring. Formal neuropsychological assessment was available in ten patients (45.5%); in the rest of the cases, testing was not feasible due to severe intellectual disability or other reasons. The mean “delay to surgery” was ten years and the mean “presurgical work-up time” was ten months.

### 3.2. Surgical Procedures

Fourteen interventions were resective (64%): temporal lobectomy (*n* = 8), extratemporal lobectomy (*n* = 1), lesionectomy with/without intra-operative ECoG (*n* = 5). Six were disconnective (27%): corpus callosotomy (*n* = 5) and posterior quadrant disconnection (*n* = 1). Two were neuromodulatory (9%): vagus-nerve stimulation (*n* = 2). See Table 2.

### 3.3. Seizure Outcomes

**Figure 2:**
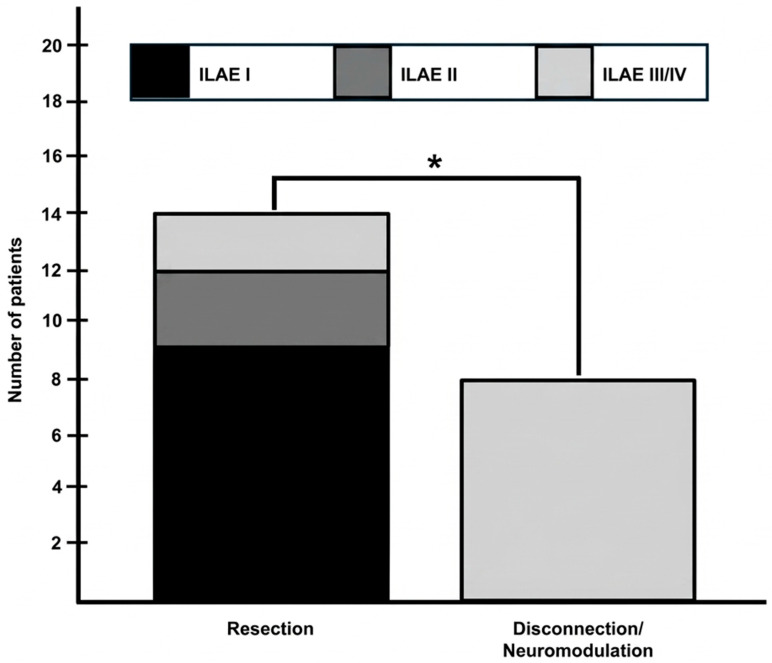
Seizure outcomes by procedure group (ILAE categories). Stacked bars show outcome distribution for resections (*n* = 14) versus disconnection/neuromodulation (*n* = 8). Note the predominance of ILAE I–II after resection (ILAE I 9/14; ILAE II 3/14) and the palliative profile in disconnection/neuromodulation (ILAE III/IV 8/8). Asterisk denotes statistical significance: Kruskal–Wallis: H = 14.9, *p* < 0.001.

At a median follow-up of 14 months (range 12–34), overall seizure-freedom rates were: ILAE I: 41% (9/22); ILAE II: 14% (3/22); ILAE III: 23% (5/22) and ILAE IV: 23% (5/22). Outcomes differed significantly by surgical procedure (H [2] = 14.9, *p* < 0.001). Resective surgery achieved ILAE I in 64% (9/14) compared with ILAE III/IV in 100% (8/8) after disconnection and neuromodulation (see Figure 2). Histopathology confirmed hippocampal sclerosis (*n* = 5), different subtypes of focal cortical dysplasia (*n* = 5), cavernoma (*n* = 2), low-grade epilepsy-associated tumors [LEATs] (*n* = 2), and gliosis (*n* = 1).

### 3.4. Complications

There were no peri-operative deaths or permanent neurological deficits. One patient who underwent corpus callosotomy developed immediate post-operative aseptic meningitis, which resolved within three days of corticosteroid and analgesic therapy without residual sequelae.

## 4. Discussion

This study aimed to describe the experience of a young Epilepsy Surgery Program launched in the context of a recently implemented epilepsy monitoring unit in the central-western region of Mexico [19]. Our cohort was young (mean 21 years), consistent with the demographic skew toward early-onset, drug-resistant epilepsy (DRE) reported by recent series in both high-income countries (HICs) and LMICs [9,20]. The male predominance (68%) could reflect a random variation in a small series. However, we cannot exclude that this also reflects referral and access inequities in underserved populations, where sociocultural and economic barriers may differentially limit timely surgical evaluation for women.

Structural etiologies were the leading cause (68%), consistent with regional and global data showing structural lesions in around 65–75% of DRE cases [2,21]. In the original etiologic breakdown, several cases were initially labeled as “genetic” based on highly suggestive electroclinical phenotypes. However, because molecular confirmation was not available (only one patient underwent a gene panel, yielding a variant of uncertain significance), we reclassified these cases as unknown etiology to avoid overinterpretation. This is consistent with current approaches in which a “genetic etiology” designation generally requires molecular support, and diagnostic yield may remain limited where next-generation sequencing is not systematically accessible [22]. Autoimmune-associated epilepsy was uncommon in our cohort (5%), in line with prospective multicenter data showing a low prevalence of neural autoantibodies among epilepsy surgery populations [23].

Standard 1.5 and 3.0 T MRI localized the presumed epileptogenic zone (EZ) in 59% of patients, in line with well-established data reporting that around one-third of patients are MRI-negative [24,25]. The added value of FDG-PET was evident; although obtained in fewer than half of patients (illustrating the cost barrier in resource-limited settings), it was concordant in 70% of studies. At first glance, this figure might appear high relative to previous studies [24], especially considering that it is reported as a pooled average. However, this value represents the yield only among the patients who actually underwent the study, those with focal epilepsy, the majority of whom had temporal lobe epilepsy. Consequently, the finding is consistent with published data showing that FDG-PET is positive and concordant in up to 80% of temporal lobe epilepsy cases [26,27].

Delay to surgery remains a major challenge. The decade-long median delay from seizure onset to surgery reproduces the sobering 8- to 12-year lag documented worldwide and underscores persistent under-referral, even in HICs where elite centers have recently trimmed the interval to ≤3 years [14,20,28,29]. In Latin American countries, sociocultural barriers, uneven insurance coverage, and limited surgical capacity prolong this odyssey; nevertheless, our timeline is modestly better than the 12- to 14-year delays described in multicenter regional surveys [30], suggesting that structured referral pathways can shorten wait times even in constrained environments.

The distribution of surgical techniques in our mixed adult-pediatric cohort mirrors what has recently been reported by other Latin-American programs [30]. Two-thirds of our operations were classic resections, mainly temporal lobectomies for mesial hippocampal sclerosis and extratemporal lobectomies for focal cortical dysplasia (FCD). A single-center Mexican series of 95 patients showed a very similar pattern, with 74% undergoing temporal lobectomy and 10% lesionectomy, while palliative corpus callosotomy constituted 16% of the caseload [31]. Region-wide survey data collected by the ILAE-Latin America Surgical Commission also place resective procedures at about 70% of all surgeries, dominated by temporal lobe cases related to hippocampal sclerosis or FCD types IIa/IIb [30]. Given that these lesions account for the bulk of drug-resistant epilepsy in both children and adults, their predominance in our series is expected and underscores the common ground between emerging and established centers in the region.

By contrast, neuromodulatory procedures were uncommon (9%), a proportion well below those reported in large North-American datasets in which vagus-nerve stimulation (VNS) alone can reach 20–25% of interventions [32,33]. The disparity is largely economic: the upfront cost of implantable stimulators in most Latin-American health systems remains a major barrier to widespread adoption. Even in high-resource settings, the high device cost has historically been cited as one of the principal obstacles to VNS deployment, a challenge that is amplified in resource-limited environments [34].

Overall seizure freedom (ILAE I) reached 41%, modest compared with pooled HIC rates of 50–70% but within the 35–55% corridor reported by recent LATAM series [5,7,8,9,30,35]. When stratified by surgical intent, curative procedures (resections and posterior disconnection) achieved 60% seizure freedom, aligning with contemporary HIC benchmarks and recent meta-analyses [36]. Conversely, purely palliative interventions such as corpus callosotomy and VNS yielded only palliative control (ILAE III/IV in 100%), consistent with their traditional role in seizure mitigation rather than complete seizure suppression [37,38]. However, the follow-up period may not fully capture the evolving seizure-reduction profile of neuromodulatory procedures, and longer longitudinal observation will be necessary to assess their true impact in this cohort. It is important to mention that, given the limited statistical power imposed by the small sample size, this comparison reflects differences in surgical intent rather than procedure effectiveness.

Our histopathological spectrum, dominated equally by hippocampal sclerosis (HS, *n* = 5) and focal cortical dysplasia (FCD, *n* = 5), mirrors patterns reported in large European and North-American surgical series [39,40,41]. In the landmark European Epilepsy Brain Bank study (*n* ≈ 9000 specimens), HS was the single most common diagnosis in adults, whereas FCD prevailed in pediatric cohorts; tumors and cavernomas together accounted for roughly 18% of cases [42]. Our combined LEATs and cavernomas represent 18% as well, suggesting that, despite treating a socio-economically constrained population, our case-mix is broadly comparable to high-income centers.

Seizure outcomes in our cohort aligned closely with the underlying pathology. All five HS cases achieved ILAE I/II, consistent with the 70–80% seizure-freedom historically associated with mesial temporal lobe resections. Conversely, outcomes for FCD were more heterogeneous (three ILAE I, two ILAE III), reflecting the well-known variability tied to FCD subtype, lesion extent, and eloquent cortex involvement [43,44,45]. Similarly, both LEAT cases reached ILAE I and ILAE III, in line with reports that show variable prognoses depending on whether complete resection is feasible or not [46,47].

Regarding safety, our single complication, an aseptic (chemical) meningitis after corpus callosotomy (CC), translates to a 4.5% overall and 16.7% CC-specific incidence [48,49]. This figure is lower than early CC series that described chemical meningitis rates of 30–50%, yet concordant with more recent data placing the risk at roughly one-third of CC patients when the ependyma is breached [50]. A systematic review of elective intracranial surgery estimated a 1.6% meningitis prevalence across all procedures, highlighting that CC carries a distinctly higher but mainly self-limited risk profile [50].

This study has several important limitations that should be considered when interpreting its findings. First, the cohort is relatively small and derives from a single center, which limits statistical power and constrains generalizability to other settings with different referral patterns and resource availability. Second, although we updated the dataset to ensure a minimum of 12 months of follow-up, longer longitudinal observation remains necessary to confirm the durability of seizure outcomes, particularly for extratemporal resections and palliative procedures where late relapse and evolving seizure phenotypes may occur. Third, etiologic classification (particularly for suspected genetic cases) was limited by restricted access to molecular testing, which may have led to misclassification within the “unknown” category and underscores the need for expanded diagnostic infrastructure. Finally, neuropsychological assessments were not uniformly available across all patients and ages, which may underestimate cognitive and psychosocial outcomes that are highly relevant to surgical decision-making and post-operative counseling. Despite these limitations, the primary aim of this work is not to establish novel predictors of outcome, but to provide real-world evidence that a structured epilepsy surgery pathway can be implemented safely and yield meaningful seizure control in a socioeconomically underserved population.

In this context, and from an implementation standpoint, we found several pragmatic strategies helpful for initiating an epilepsy surgery pathway in a resource-constrained environment. First, we performed a case-by-case risk assessment for status epilepticus to determine whether pre-admission antiseizure medication reduction was safe, thereby optimizing the likelihood of capturing habitual seizures during the EMU stay while preserving patient safety. Second, we actively requested smartphone home videos from families to refine semiologic characterization in advance. For example, in highly stereotyped events, capturing a single representative seizure during inpatient video-EEG was often sufficient to proceed to multidisciplinary discussion. Third, we adopted a “good-enough imaging” principle. This means that, when patients arrived with an acceptable-quality lesional MRI, even without a dedicated epilepsy protocol, we used it to avoid delays and reserved repeat imaging for cases with uncertainty. Fourth, for out-of-town patients with limited ability to return, we coordinated neuropsychological assessment early, frequently initiating testing during EMU admission. Fifth, we used a tiered diagnostic approach, standardizing video-EEG and MRI as the minimum dataset and escalating to PET, fMRI, or additional testing only when strictly necessary and feasible for the individual patient. Finally, we held virtual multidisciplinary epilepsy surgery conferences, enabling participation of national and international colleagues to strengthen decision-making without increasing local infrastructure demands.

Our data support recommendations from the WHO Intersectoral Global Action Plan calling for integration of epilepsy surgery into national health strategies for drug-resistant epilepsy. Priority actions should focus on shortening referral delays, decentralizing presurgical evaluation through telemedicine, and establishing financing mechanisms to subsidize surgical care for underserved populations.

## 5. Conclusions

In conclusion, timely resective surgery offers high seizure-freedom rates even in low-resource settings, but systematic efforts are required to reduce the diagnostic-to-treatment interval and close the epilepsy-surgery gap.

## Figures and Tables

**Table 1:** Baseline characteristics and presurgical evaluation of the study cohort (*n* = 22).

Characteristic	Value
Demographics	
Age at surgery, years	21.2 ± 11.2
Sex, male	15 (68%)
Sex, female	7 (32%)
Epilepsy classification	
Epilepsy type—focal	15 (68%)
Epilepsy type—generalized/multifocal	7 (32%)
Etiology	
Structural	15 (68%)
Genetic	6 (27%)
Autoimmune	1 (5%)
Presurgical investigations	
Long-term video-EEG completed (≥2 habitual seizures)	21 (95%)
MRI performed	22 (100%)
MRI concordant with presumed EZ	13 (59%)
FDG-PET performed	10 (45.5%)
FDG-PET concordant with presumed EZ	7 (70%)
Neuropsychological Assessment	10 (45.5%)
Time metrics	
Delay from epilepsy onset to surgery, years (mean, IQR)	10 (8–15)
Presurgical work-up time, months (mean, IQR)	10 (6–15)
Follow-up duration, months. Mean (interval)	14 (12–34)

Concordance is agreement between test findings and the presumed epileptogenic zone (EZ) as defined by the multidisciplinary team and confirmed by post-surgical outcome. Abbreviations: MRI = magnetic resonance imaging; FDG-PET = ^18^F-fluorodeoxyglucose positron emission tomography; EEG = electroencephalogram.

**Table 2:** Surgical procedures and seizure outcomes by ILAE class.

Procedure	*n* (% of Cohort)	ILAE I *n* (%)	ILAE II *n* (%)	ILAE III *n* (%)	ILAE IV *n* (%)	Complication *n* (%)	Complication Type (If Any)
Resective procedures							
Temporal lobectomy	8 (36.4%)	5 (62.5%)	2 (25%)	1 (12.5%)	0	0	—
Extratemporal lobectomy	1 (4.5%)	1 (100%)	0	0	0	0	—
Lesionectomy (w/o intra-operative ECoG)	5 (22.7%)	3 (60%)	1 (20%)	1 (20%)	0	0	—
Disconnective procedures							
Corpus callosotomy	5 (22.7%)	0	0	2 (40%)	3 (60%)	1 (20%)	Aseptic (chemical) meningitis
Posterior quadrant disconnection	1 (4.5%)	0	0	1 (100%)	0	0	—
Neuromodulatory procedures							
Vagus-nerve stimulation	2 (9.1%)	0	0	0	2 (100%)	0	—
Overall							
Overall (all procedures)	22 (100%)	9 (40.9%)	3 (13.6%)	5 (22.7%)	5 (22.7%)	1 (4.5%)	Aseptic (chemical) meningitis

Percentages in ILAE columns use the procedure-specific n as denominator; the “% of cohort” column uses *n* = 22. Abbreviations: ECoG = electrocorticography; ILAE = International League Against Epilepsy.

## Data Availability

The datasets generated during and/or analyzed during the current study are available from the corresponding author upon reasonable request due to patient privacy and confidentiality constraints.
